# Normal Lung Tissue CT Density Changes after Volumetric-Arc Radiotherapy (VMAT) for Lung Cancer

**DOI:** 10.3390/jpm12030485

**Published:** 2022-03-17

**Authors:** Marek Konkol, Maciej Bryl, Marek Fechner, Krzysztof Matuszewski, Paweł Śniatała, Piotr Milecki

**Affiliations:** 1 Electroradiology Department, Poznan University of Medical Sciences, 61-866 Poznan, Poland; piotr.milecki@wco.pl; 2 IV Radiotherapy Department, Greater Poland Cancer Center, 61-866 Poznan, Poland; 3 Oncology Department, Greater Poland Center of Pulmonology and Thoracic Surgery, 60-569 Poznan, Poland; mbryl@wcpit.pl; 4 Independent Researcher, 60-965 Poznan, Poland; marek.fechner@gmail.com; 5 Medical Physics Department, Greater Poland Cancer Center, 61-866 Poznan, Poland; krzysztof.matuszewski@wco.pl; 6 Institute of Computing Science, Poznan University of Technology, 60-965 Poznan, Poland; pawel.sniatala@put.poznan.pl; 7 I Radiotherapy Department, Greater Poland Cancer Center, 61-866 Poznan, Poland

**Keywords:** radiation-induced lung injury, density changes, radiodensity, radiation pneumonitis, radiotherapy, lung cancer

## Abstract

Radiation-induced lung injury remains a significant toxicity in thoracic radiotherapy. Because a precise diagnosis is difficult and commonly used assessment scales are unclear and subjective, there is a need to establish quantitative and sensitive grading methods. The lung tissue density change expressed in Hounsfield units (HUs) derived from CT scans seems a useful numeric surrogate. The study aimed to confirm a dose-response effect on HU value changes (ΔHU), their evolution in time, and the impact of selected clinical and demographic factors. We used dedicated, self-developed software to register and analyze 120 pairs of initial and follow-up CT scans of 47 lung cancer patients treated with dynamic arc radiotherapy. The differences in HU values between CT scans were calculated within discretized dose-bins limited by isodose lines. We have proved the dose-effect relationship, which is well described with a sigmoid model. We found the time evolution of HU changes to suit a typical clinical presentation of radiation-induced toxicity. Some clinical factors were found to correlate with ΔHU degree: planning target volume (PTV), V35 in the lung, patient’s age and a history of arterial hypertension, and initial lung ventilation intensity. Lung density change assessment turned out to be a sensitive and valuable method of grading post-RT lung toxicity.

## 1. Introduction

Radiation-induced lung injury (RILI) remains one of the most clinically significant toxicities in thoracic radiotherapy (RT), irrespective of modern delivery techniques. Thus, a complete understanding of underlying mechanisms, predictive factors, and assessment methods is crucial.

The early, subacute inflammatory response is known as radiation-induced pneumonitis (RIP) and usually occurs within the first three months after treatment (1–6 months). It is related to the infiltration of immune effector cells like neutrocytes, monocytes, macrophages, and the release of proinflammatory cytokines and chemokines. Later profibrogenic and proangiogenic stimulation leads to the fibrotic phase called radiation-induced lung fibrosis (RILF) [1]. Presentations of RIP vary from asymptomatic radiographic findings to life-threatening conditions. The most common symptoms are non-productive cough, dyspnea (mild to severe), moderate fever, and general fatigue. If the volume of the injured lung is extensive enough, RIP might carry to respiratory failure. Advanced RILF presents as progressive dyspnea, developing pulmonary hypertension with right-heart failure, and a possible fatal scenario [2]. There is a whole spectrum of radiation-induced image changes. The earliest findings, even 3–4 weeks after the RT, are mainly ground-glass and reticular opacities progressing to airspace mass-like and scar-like consolidations and traction bronchiectasis. All these changes can resolve entirely within weeks [3]. However, some patients can propagate to late fibrosis radiologically manifested as sharply defined consolidation or linear scarring with volume loss and architectural distortion [4].

Because of a diverse clinical-radiological presentation, often overlapping with other comorbidities, and the difficulty of precise diagnosis, a broad range of RIP incidence rates can be found in the literature and vary from 5% to 58% [2]. For any symptomatic pneumonitis (grade 2 or higher), values of 30–35% are reported for static 3D radiotherapy and 24–34% for intensity-modulated RT (IMRT)/volumetric-arc techniques (VMATs) [5]. Nevertheless, modern radiotherapy methods significantly decrease the highest grades of toxicity. In a recent trial with stage III NSCLC patients who qualified for durvalumab consolidation, the incidence of G3+ pneumonitis was 7% (34% for G2+) [6]. Similarly, Hu et al. [7] reported G3+ RIP in 1% of patients and Shintani et al. [8] 5% for G3+ (35% for G2+). Late RILF is less frequently reported, and for IMRT is around 30% (G1 and single cases of G3+) [7].

There are different clinical scales to assess radiation-induced lung toxicity. The most used are CTCAE v5.0 (Common Terminology Criteria for Adverse Events, version 5.0), LENT-SOMA (Late Effects in Normal Tissue—Subjective Objective Management Analysis), RTOG (Radiation Therapy Oncology Group), and SWOG (Southwest Oncology Group). However, differences between the scales and their unclear, subjective definitions hamper comparison of research results, especially in low-grade, subclinical patients.

Hence, there is a need to establish quantitative and sensitive methods of lung injury assessment. There are attempts to use radiomics: image-based objective features. The lung tissue density change expressed in Hounsfield units (HUs) derived from CT scans can be a numeric surrogate as it seems to fit the most implemented NTCP models of lung response to radiation [9]. Some authors showed a dose-response relation, evolution in time, and basic predictive factors [10,11,12,13,14,15]. There are few examples of dedicated software for big-data analysis of DICOM (Digital Imaging and Communications in Medicine) lung images [16].

The aim of this study was to confirm a dose-response effect on HU value changes, their evolution in time, and the impact of selected clinical and treatment-related factors.

## 2. Materials and Methods

### 2.1. Patient Characteristics

Data from 47 lung cancer patients treated between January 2015 and October 2017 in Greater Poland Cancer Center, Poznan, Poland, were retrospectively analyzed. All the patients underwent VMAT (volumetric arc technic) radiotherapy on Varian Clinac and TrueBeam accelerators with a total dose range between 30 Gy and 66 Gy in 2- or 3-Gy fractions. All doses were recalculated as 2 Gy equivalents (EQD2, α/β = 3 Gy) [17]. Most of them (37 pts) received sequential chemotherapy, mainly platinum-based. Treatment plans were calculated using Eclipse Planning Software v13 (Varian Medical Systems Inc., Palo Alto, CA, USA). Radiotherapy plans met restrictive dose-constraints: mean lung dose (MLD) < 16 Gy, V20_lung < 20% (the percentage of lung volume receiving ≥ 20 Gy), V5_lung < 70%. In total, 120 follow-up CT scans had been performed in different time intervals (52–754 days after RT completion). None of the patients had an intra-pulmonary recurrence observed. Medical records concerning only two patients suggest a possible Grade 2 pneumonitis (cough, dyspnea) during the post-treatment period; however, precise data are lacking. Details are given in Table 1.

### 2.2. Software Workflow and Data Analysis

We have developed a dedicated server-based application described in [16] for study purposes. The workflow started with uploading CT images and RT-specific DICOM files (RT_dose, RT_structure) and automatically selecting lung-containing scans using artificial intelligence (neural network VGG16). After setting up the parameters, the analysis began with lung segmentation, proceeding to affine and elastic registration. Finally, the HU value differences (ΔHU) between geographically corresponding voxels on both series were calculated. The volume of interest was limited by a defined dose range minus planning target volume (PTV). Each time, the patient’s initial CT scans obtained during RT planning (iRT scans) were compared to the consecutive follow-up ones (FU scans), matched into 3- and 6-month intervals (0–3, 3–6, 6–9, 9–12, 12–18, over 18 months post-RT). Contours were created from the isodose lines to discretize analysis into 5- and 10-Gy dose bins (0–5, 5–10, 10–20, 20–30, 30–40, 40–50, 50–60 Gy).

Similarly to other studies, a minor density change at low doses was assumed, and for each patient, the mean ΔHU in the lowest dose bin (lung voxels receiving < 5 Gy) was subtracted from the mean ΔHU of all other dose bins to offset the differences between CT scanner calibrations.

The dose-response sigmoids were fitted to the obtained data points according to the following logistic function, as in Defraene et al.’s study [12]:

(1) $$\Delta \mathrm{HU}=\frac{\Delta \mathrm{HUmax}}{1+e^{4\gamma \;\left(1-\frac{D}{D50}\right)}}$$

where ΔHUmax is the ΔHU maximum level, D_50_ and D_95_ are the doses at which 50% and 95% of ΔHUmax is reached, respectively, and γ is the steepness of the sigmoid. The sigmoids were fitted using the generalized reduced gradient (GRG) method within the Solver add-on in Excel (Microsoft Excel for Mac, Version 16.56), employing an iterative least-squares fitting routine to achieve the optimal goodness of fit between data and function. Besides the dose-response curves, the time evolution of ΔHU in the individual dose-bins was presented.

Apart from a standard analysis comparing iRT vs. FU scans, an initial 4DCT ventilation assessment was performed. To determine the ΔHUvent values, CT scans of the outermost respiratory phases (phases 0% and 50%, obtained during 4DCT for the RT planning) were registered and analyzed as above.

The following patient- and treatment-related factors’ impact on density change were investigated: age and sex, preceding surgery, tumor pathology type, comorbidities (diabetes, COPD/asthma, arterial hypertension (AH)), concomitant chemotherapy and dose distribution parameters (PTV volume, V5_lung, V10_lung, V20_lung, V35_lung, MLD, maximum heart dose (MHD), mean heart dose (MeanHD), V5_heart, V40_heart, V45_heart, V50_heart).

The Wilcoxon matched paired test was used to determine the significance of HU increase in relation to dose and time. Pearson’s r and Spearman’s rank correlation coefficients were used to test the influence of the above-mentioned clinical data, and Student’s *t*-test was performed for subgroup comparison. Two-tailed *p*-values less than 0.05 were considered statistically significant. Error bars in the figures represent 95% confidence intervals. The PQStat v1.8.2.230 (PQStat Software, Poznań, Poland) was used.

## 3. Results

### 3.1. Lung Density Changes in the Function of Dose

**Figure 1:**
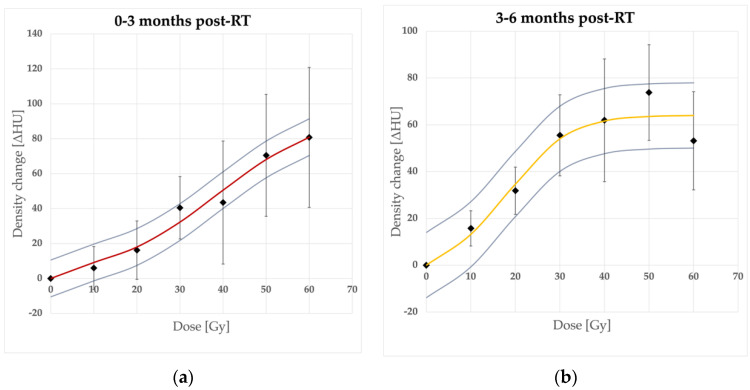

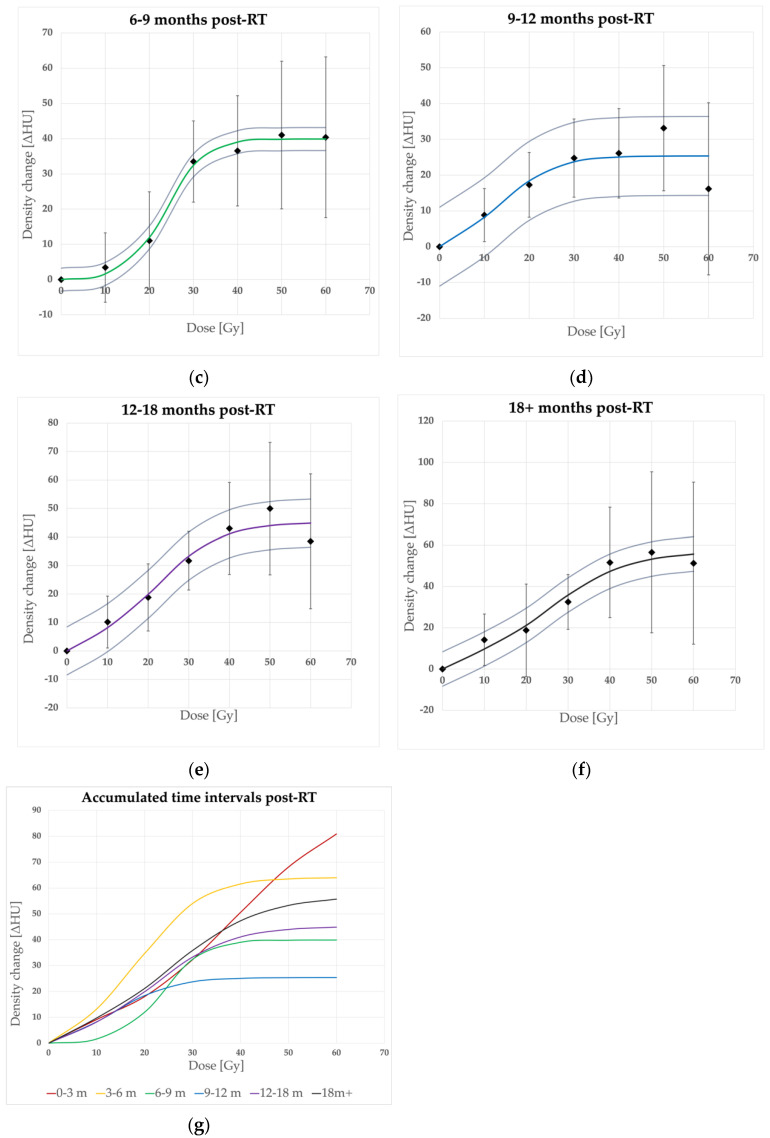
Dose-response sigmoidal model fit in consecutive time intervals. Density changes in time intervals after RT completion: (**a**) 0–3 months, (**b**) 3–6 months, (**c**) 6–9 months, (**d**) 9–12 months, (**e**) 12–18 months, (**f**) over 18 months, and (**g**) accumulated curves. 95% CI bars (for mean ΔHU data points) and curves (for models)—omitted in (**g**) for clarity.

The lung density, expressed by the HU values, increased significantly (*p* < 0.05) compared to the baseline values for all time and dose intervals, apart from 5–10 Gy and 10–20 Gy ranges in the earliest phase—0–3 months (*p* = 0.233435, *p* = 0.077162, respectively) and 5–10 Gy and 10–20 Gy ranges in 6–9 months (*p* = 0.701978, *p* = 0.167319). The mean HU response was well described sigmoidally with a presumed model (Figure 1) as the data points within consecutive time intervals fit the logistic function (Equation (1)) rightly. Maximum ΔHU ranged from 25.4 (14.4–36.4) HU to 96 (85.5–106.5) HU. The dose points at which the saturation of HU changes is reached (D_95_) differed between time intervals. Our model estimates started from 70.2 Gy for the earliest changes and decreased to 38.4–31.6 Gy for 3–6, 6–9, and 9–12 month periods to increase after 12 months to 45.1–53 Gy.

The model parameters are summarized in Table 2. These findings confirm the dose-response relation in radiodensity changes.

### 3.2. Time Evolution of Lung Density Changes

**Figure 2:**
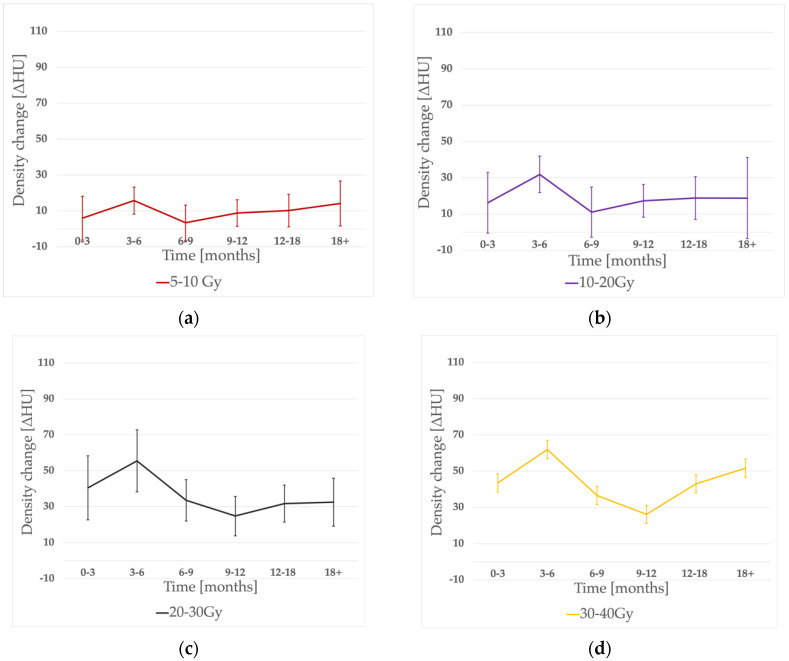

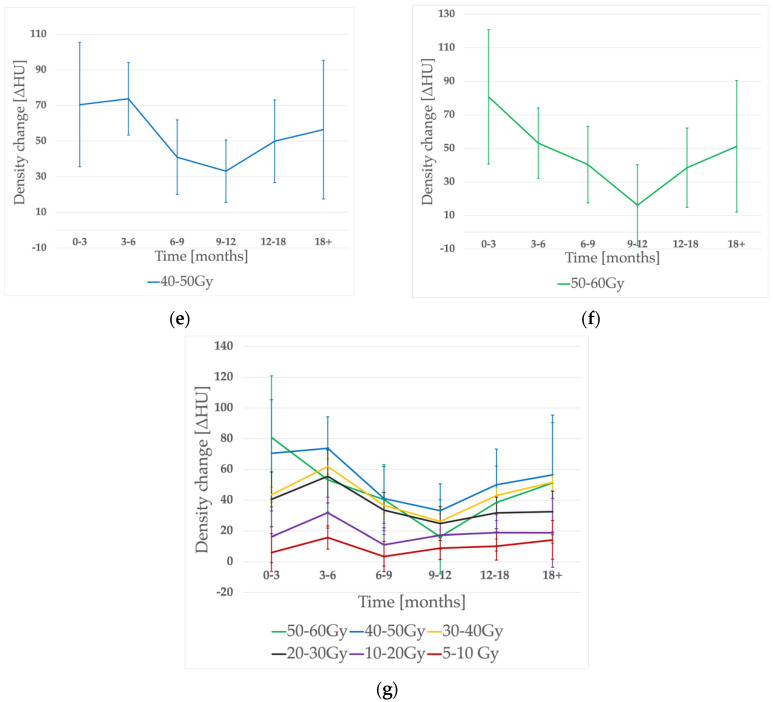
Density change evolution in time for different dose-bins: (**a**) 5–10 Gy, (**b**) 10–20 Gy, (**c**) 20–30 Gy, (**d**) 30–40 Gy, (**e**) 40–50 Gy, (**f**) 50–60 Gy, and (**g**) accumulated graph.

The time evolution of density changes is shown in Figure 2. As expected, the noticeable peak of ΔHU was seen within 3–6 months post-RT in almost all of the dose-bins (besides the 50–60 Gy range, when it occurred earlier). However, there was no significant difference between the 0–3 and 3–6 months periods (*p* = 0.43). Then, a significant (*p* = 0.03) decrease in density occurred, reaching its nadir at 6–9 months for lower doses (5–10 Gy, 10–20 Gy bins) and 9–12 months for the rest of the dose ranges. A consecutive, significant (*p* = 0.03) dose increase was observed in the whole dose range. The earlier ΔHU peak was significantly higher than the later one (*p* = 0.036).

### 3.3. Factors Influencing Density Changes Degree

#### 3.3.1. Clinical Factors Influencing the Early Phase (3–6 Months)

We found a few clinical variables to have a significant impact on radiodensity during the early phase corresponding to radiation pneumonitis. The PTV volume was significantly, positively correlated with ΔHU in mid-dose areas receiving 20–30 Gy (*p* = 0.000288, r = 0.625, r^2^ = 0.390) and 30–40 Gy (*p* = 0.029412, r = 0.404, r^2^ = 0.163). We also found V35_lung to correlate with HU change intensity in 20–30 Gy and 30–40 Gy areas (*p* = 0.025656, r = 0.41, r^2^ = 0.171; *p* = 0.004156, r = 0.3516, r^2^ = 0.266, respectively). For higher doses (40–50 Gy), patients’ age turned out to impact ΔHU (*p* = 0.049, r = 0.397, r^2^ = 0.157). Patients who had had surgery before RT presented a more pronounced HU increase within a 5–10 Gy area (*p* = 0.015465) and those with a history of arterial hypertension had intensified HU increase within 40–50 Gy and 50–60 Gy levels (*p* = 0.003208, *p* = 0.02017).

#### 3.3.2. Clinical Factors Influencing the Late Phase (over 12 Months)

For late changes, we observed that density increased more in older patients. The linear correlation was noted between the ΔHU and patient’s age for 20–30 Gy, 30–40 Gy, and 40–50 Gy dose-bins (*p* = 0.01122, r = 0.518, r^2^ = 0.269; *p* = 0.007244, r = 0.555, r^2^ = 0.308; *p* = 0.005905, r = 0.592, r^2^ = 0.351, respectively). For mid-dose ranges (20–30 Gy, 30–40 Gy), patients with preceding surgery (*p* = 0.0348; *p* = 0.0111) and a history of AH (*p* = 0.0051; *p* = 0.0188) had an intensified ΔHU response.

#### 3.3.3. DCT Ventilation

First, we assessed the aeration of lung sub-volumes measuring the radiodensity differences (ΔHU_vent) between 0 and 50% ventilation phases on initial 4DCT scans. The ΔHU_vent values ranged from 0.08 to 59.03 HU (mean 17.89 HU). We found that the higher the ΔHU_vent, the more pronounced the density changes in low-dose volumes. For the early 3–6 month period there was a correlation within 5–10 Gy and 10–20 Gy dose-bins (*p* = 0.035, r = 0.461; 0.014, r = 0.49) and for the late one (over 12 months) the correlation was seen for 10–20 Gy (*p* = 0.048, r = 0.435).

## 4. Discussion

As far as we know, this is one of the most complex studies assessing radiodensity changes in lung cancer patients treated with modern, VMAT-only techniques with conventional fractionation in both radical and palliative settings.

The most important part concerning the dose-response relation confirmed its existence and a possible description by a sigmoid function proposed by Defraene et al. [12]. Their results concerning a slightly larger group of NSCLC patients in one follow-up period only (3 months) seem to be in line with ours (ΔHU_max = 92.9 HU vs. 96 HU, D_50_ = 29.9 Gy vs. 38.6 Gy, γ = 0.74 vs. 0.76). Similarly, in a study by Bernchou et al. [11], maximum density changes reached the level of 60–90 HU. In the SBRT study by Palma et al. [14] most of the patients presented a ΔHU within the 0–100 HU range, except for those with a PTV > 100 m^3^, where the HU changes up to 150 HU were observed.

The novelty and precision of our computer-aided method is worth underlining, as it is difficult to see subtle changes within 80–100 HU with the naked eye using a typical, wide “lung-window” (width: 1500 HU) and these are hardly ever described in routine CT examination. What is more, these changes were noted despite the patients being mostly asymptomatic, and the restrictive dose-constraints for radiotherapy plans being met. The threshold value for symptomatic lung damage probably starts within the end of this range. Diot et al., for example, used a ΔHU > 80 HU as a subjective threshold to be associated with a moderate radiologic physician-graded lung injury that practically fell between Radiation Therapy Oncology Group grades 1 and 2 if the determination was purely based on radiographic findings [18].

In the earliest period, the HU saturation dose point (D_95_) was noticeably higher (70.2 Gy) than for later ones. This can be related to possible geographical inaccuracy of registration in 50–60 Gy dose areas, closest to PTV, in these patients. For the rest of the time intervals, D_95_ was between 30 and 50 Gy, which seems to be lower than in other studies. That might be because of a lower mean prescribed dose in our study (we have included both radical 60–66 Gy patients and 30 Gy palliative ones). Nevertheless, we can assume (confirming the Defraene results) that potential dose-escalation over standard radical doses would have a negligible effect on radiodensity.

The time evolution of ΔHU corresponds with a typical clinical scenario. We observed the maximum ΔHU within the 0–3 and 3–6 month periods, which matches the typical time of radiation pneumonitis usually observed between 1–6 months post-RT [19]. The subsequent significant decrease occurred during the possible healing period, called the intermediate phase, when the migration of fibroblasts restores the tissue integrity and hyaline membranes are dissolute [5]. Finally, the intensifying hypoxia promotes further profibrogenic and proangiogenic stimulation that leads to the fibrotic phase (9 months after RT); hyperplastic pneumocytes and myofibroblasts and collagen deposits in the lung interstitium can be observed. That corresponds with our findings describing the significant increase in density after the 9–12 month time-point. The new peak was significantly less pronounced than the early one and seemed to reach its plateau after 12–18 months. Similar time evolution was described by Bernchou et al. [11], who proposed a two-component model, separating early pneumonitis from late fibrosis as distinct phenomena. However, we observed an even more sharply expressed HU decrease within 6–12 months during the healing period, which could confirm the time-shift and separation of both processes. It is also worth underlining that our follow-up CT scans had been obtained in more scattered time points within the proposed time intervals than other studies with strictly specified time-points, which could influence the results.

Because of relatively small subgroups of patients, it was difficult to point out many clinical factors impacting on density change and carry out a complex multivariable analysis. Nevertheless, we managed to find some possible predictors. For the early phase, within the mid-dose region (20–40 Gy), we noticed a linear correlation between ΔHU and PTV volume and, as a logical consequence, between ΔHU and the V35_lung parameter. The irradiated volume has already been described as a risk factor for radiation pneumonitis [14,20], whereas the V35_lung does not have a strong literature background yet. We did not find any correlation with the most commonly used parameters like V5, V20, or MLD, probably because of very restrictive dose-constraints for thoracic plans in our institution (average MLD = 10.3 Gy, V20_lung < 17.2%). Some suggest heart doses to be even more critical. The parameters with the strongest correlation with G2+ pneumonitis in the literature are the mean heart dose (MHD), V65, and V43 [21,22,23]. Although a simple explanation is missing, we can suspect that radiation-induced right-heart dysfunction can promote pulmonary hypertension, oedema, and transudate. There was no correlation with these parameters in our patients, possibly because of low heart doses on our plans (the average mean heart dose—7.8 Gy).

We found that the patients’ age correlates positively with the intensification of density changes on both early follow-up scans (40–50 Gy dose-bins) and those after 12 months (20–50 Gy bins). It was somehow expected, as some authors point out an older age (65+) as a potential risk factor of RIP [20,24,25]. This relation can result from numerous comorbidities, which are independent risk factors. However, this assumption is not clear when analyzing the most extensive trials—probably with the most precise selection of patients [6]. Another controversial finding concerns the preceding surgery, which we noticed linked with intensified HU changes during the early phase in low-doses (5–10 Gy) and mid-doses (20–40 Gy) for late fibrosis. Surgery is generally reported to be unrelated to RIP [25]. However, some authors point to a possible correlation with radiation-induced toxicity that can be observed despite low V20, MLD, and MHD values in postoperative radiotherapy [21]. Finally, we showed that patients with a history of hypertension had significantly more pronounced lung density changes in both early (3–6 months) and late (12 months+) post-treatment scans in mid- and high-dose volumes. There is no specific literature data on that risk factor itself. We can only speculate that this is the equivalent of general cardiovascular comorbidity in our study; however, the precise medical data on cardiological assessment of our patients were unavailable. Diabetes mellitus and COPD/asthma were not found to impact radiodensity. The fact of receiving sequential chemotherapy or its type was also insignificant. This was expected, as most of our patients received platinum-based schemes, which are considered unrelated to RIP. Well-known agents that increase the risk of RIP are taxanes, doxorubicin, bleomycin, cyclophosphamide, vincristine, mitomycin, gemcitabine, recombinant interferon alfa, and bevacizumab [26]. Because of the synergistic effect with radiotherapy, they possibly act like radiosensitizers. Exceptionally high risk is reported for paclitaxel-based chemotherapy [2].

The software we have developed lets us assess the ΔHU_vent between CT scans of the outermost respiratory phases on initial 4DCT. We have assumed this parameter to be a surrogate of ventilation degree as the density reflects the percentage of air in measured volume. Similar 4DCT-derived density changes have already been investigated, e.g., Palma et al. and others [27,28]. Our findings revealed a positive correlation between ΔHU_vent and ΔHU for low doses in both the early and late phases. That could potentially be explained by the oxygen enhancement on tissue exposed to low doses. Within the higher doses, the radiation dose is a strong enough factor that the effect of ventilation is blurred. The CT-derived ventilation measurement has become increasingly utilized in radiation oncology for functional avoidance radiotherapy, where the radiation plan is designed to avoid the most functional portions of the lungs because its damage can cause the most significant functional decrease [29,30]. The next step is to link ventilation and lung perfusion, which is also possible with a non-contrast CT scan [31].

Our study also had some limitations. First, because of its retrospective nature. The time-points of follow-up CT scans were dispersed as well as follow-up visits. In many cases, we also lacked precise clinical information concerning symptoms of possible radiation pneumonitis/extensive fibrosis and current clinical assessment of comorbidities. Secondly, the group of patients was relatively small, which hampered precise subgroup analysis. We must also be conscious of the inaccuracy of the registration process. Despite modern rigid and elastic “home-tuned” algorithms, there was a possibility of geographical mismatch, especially in the areas of the greatest lung motion or closest to the PTV/GTV where the tumor and its regression leads to the most pronounced architectural distortion.

Despite all the limitations, radiodensity changes expressed in HU values seem to be a valuable method for an objective, numerical assessment of lung injury. Nevertheless, further prospective research and a complex correlation with clinical factors are needed.

## Figures and Tables

**Table 1:** Patients’ and treatment characteristics.

Patients’ Characteristics		Treatment Characteristics	
Total number of pts.	47	Mean total dose (Gy)	57 (30–66)
Median age	66 (52–82)	Fraction dose (2 Gy/3 Gy)	34/13
Women/men	20/27	Mean PTV volume (cm^3^)	217 (47–645)
NSCLC/SCLC	43/4	Mean V5_lung (%)	46 (12–71.7)
Preceding surgery (yes/no)	13/34	Mean V20_lung (%)	17.2 (4.6–31)
Sequential chemo (yes/no)	37/10	Mean lung doses (Gy)	10.36 (3.4–16.5)
Chemo type	PN (27), KN (4), PE (3), KE (2), NVB mono (1)	Mean V5_heart (%)	37.43 (0–93.5)
Total number of follow-up CTs	120	Mean V40_heart	3.53 (0–14.8)
Time distribution of follow-up CTs ^1^	0–3 m (12), 3–6 m (30), 6–9 m (18), 9–12 m (26), 12–18 m (22), 18 m+ (11)	Mean heart doses (Gy)	7.86 (0.124–23)
Comorbidities ^2^	COPD (10), AH (24), DM (8)		
Rad. pneumonitis ^3^	G1 (0), G2 (2), G3+ (0)		

^1^ m = months, ^2^ COPD—chronic obstructive pulmonary disease, AH—arterial hypertension, DM—diabetes mellitus, ^3^ radiation pneumonitis according to medical records.

**Table 2:** Parameters ΔHU_max_, D_50_, D_95_, γ, and R^2^ of the sigmoidal fit to achieved data points.

Time from RT	ΔHU_max_ (HU)	D_50_	D_95_	γ	R^2^
0–3 months	96 (85.5–106.5)	38.6 Gy	70.2 Gy	0.76	0.98
3–6 months	64.1 (50.1–78)	18.9 Gy	38.4 Gy	0.71	0.94
6–9 months	39.9 (36.6–43.1)	23.7 Gy	36.4 Gy	1.36	0.99
9–12 months	25.4 (14.4–36.4)	14.3 Gy	31.6 Gy	0.61	0.80
12–18 months	45.2 (36.7–53.7)	21.9 Gy	45.1 Gy	0.69	0.96
Over 18 months	57.1 (48.8–65.4)	25.1 Gy	53 Gy	0.66	0.97

## Data Availability

The clinical study report and deidentified individual participant data are available upon request, after approval by the study principal investigator (corresponding author) and institution authorities. All the requests will be reviewed on a case-by-case basis. In the case of approval, a specific agreement between the sponsor and the researcher might be required for data transfer.
